# Thin-slice T_2_-weighted images and deep-learning-based super-resolution reconstruction: improved preoperative assessment of vascular invasion for pancreatic ductal adenocarcinoma

**DOI:** 10.1186/s13244-025-02022-5

**Published:** 2025-06-30

**Authors:** Xiaoqi Zhou, Yuxin Wu, Yanjin Qin, Chenyu Song, Meng Wang, Huasong Cai, Qiaochu Zhao, Jiawei Liu, Jifei Wang, Zhi Dong, Yanji Luo, Zhenpeng Peng, Shi-Ting Feng

**Affiliations:** Department of Radiology, The First Affiliated Hospital of Sun Yat-sen University, Guangzhou, P.R. China

**Keywords:** Pancreatic ductal adenocarcinoma, Vascular invasion, Super-resolution, MRI, T_2_WI

## Abstract

**Purpose:**

To evaluate the efficacy of thin-slice T_2_-weighted imaging (T_2_WI) and super-resolution reconstruction (SRR) for preoperative assessment of vascular invasion in pancreatic ductal adenocarcinoma (PDAC).

**Methods:**

Ninety-five PDACs with preoperative MRI were retrospectively enrolled as a training set, with non-reconstructed T_2_WI (NRT_2_) in different slice thicknesses (NRT_2_-3, 3 mm; NRT_2_-5, ≥ 5 mm). A prospective test set was collected with NRT_2_-5 (*n* = 125) only. A deep-learning network was employed to generate reconstructed super-resolution T_2_WI (SRT_2_) in different slice thicknesses (SRT_2_-3, 3 mm; SRT_2_-5, ≥ 5 mm). Image quality was assessed, including the signal-to-noise ratio (SNR), contrast-to-noise ratio (CNR), and signal-intensity ratio (SIR_t/p_, tumor/pancreas; SIR_t/b_, tumor/background). Diagnostic efficacy for vascular invasion was evaluated using the area under the curve (AUC) and compared across different slice thicknesses before and after reconstruction.

**Results:**

SRT_2_-5 demonstrated higher SNR and SIR_t/p_ compared to NRT_2_-5 (74.18 vs 72.46; 1.42 vs 1.30; *p* < 0.05). SRT_2_-3 showed increased SIR_t/p_ and SIR_t/b_ over NRT_2_-3 (1.35 vs 1.31; 2.73 vs 2.58; *p* < 0.05). SRT_2_-5 showed higher CNR, SIR_t/p_ and SIR_t/b_ than NRT_2_-3 (*p* < 0.05). NRT_2_-3 outperformed NRT_2_-5 in evaluating venous invasion (AUC: 0.732 vs 0.597, *p* = 0.021). SRR improved venous assessment (AUC: NRT_2_-3, 0.927 vs 0.732; NRT_2_-5, 0.823 vs 0.597; *p* < 0.05), and SRT_2_-5 exhibits comparable efficacy to NRT_2_-3 in venous assessment (AUC: 0.823 vs 0.732, *p* = 0.162).

**Conclusion:**

Thin-slice T_2_WI and SRR effectively improve the image quality and diagnostic efficacy for assessing venous invasion in PDAC. Thick-slice T_2_WI with SRR is a potential alternative to thin-slice T_2_WI.

**Critical relevance statement:**

Both thin-slice T_2_-WI and SRR effectively improve image quality and diagnostic performance, providing valuable options for optimizing preoperative vascular assessment in PDAC. Non-invasive and accurate assessment of vascular invasion supports treatment planning and avoids futile surgery.

**Key Points:**

- Vascular invasion evaluation is critical for the surgical eligibility of PDAC.
- SRR improved image quality and vascular assessment in T_2_WI.
- Utilizing thin-slice T_2_WI and SRR aids in clinical decision making for PDAC.

**Graphical Abstract:**

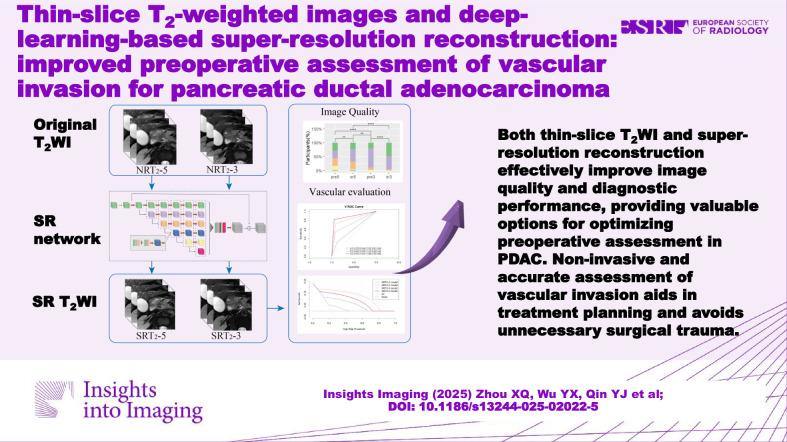

## Introduction

Pancreatic ductal adenocarcinoma (PDAC) stands out as one of the most aggressive and lethal malignancies. The estimated 5-year overall survival rate after diagnosis is typically less than 10% [1]. Among the various treatment modalities available for PDAC, radical surgical resection remains the most effective and the only potentially curative method, offering the possibility of long-term survival [2]. However, tumor invasion into major blood vessels renders many PDAC patients ineligible for surgery. Vascular invasion is a critical factor in determining preoperative resectability and serves as an important predictor of prognosis for PDAC patients [3, 4].

Preoperative non-invasive imaging techniques, such as MRI and CT, are commonly employed in clinical practice to assess vascular invasion. While CT provides better spatial resolution, MRI offers superior soft tissue contrast and is more effective at distinguishing between fibrotic or inflammatory adhesions and true vascular invasion. However, there is ongoing debate regarding significant differences in the diagnostic capabilities of CT and MRI for assessing vascular invasion. The current evidence remains insufficient, making precise evaluation of vascular involvement challenging [5]. This limitation increases the risk of positive surgical margins (R1 resection) and may lead to some inoperable patients undergoing unnecessary exploratory laparotomies.

Recent advancements in MRI technology have significantly increased spatial resolution and improved the diagnostic accuracy of high-resolution thin-slice T_2_-weighted imaging (T_2_WI) MRI for assessing vascular invasion. But such a scanning protocol requires longer scanning times. Additionally, the super-resolution technique, aiming at recovering higher spatial resolution of digital images from lower-resolution observations, has also achieved superior performance in medical imaging with the development of deep learning (DL). The rationale behind super-resolution reconstruction (SRR) is to denoise the images, enhance edges, and increase sharpness. The integration of DL-based SRR during scanning allows for fast and high-quality imaging of the pancreas, rectum, and prostate [6–8]. Furthermore, post-processing using SRR, independent of examination equipment and parameters, has been widely applied in various diseases. In the chest MRI, SRR can improve lesion detectability and enhance diagnostic confidence [9]. It can also enhance image sharpness and improve depiction of nerves and vessels in brain MRI [10]. A recent study used DL-based SRR to improve the quality of CT images for PDAC, enhancing the depiction of all structures relevant for PDAC evaluation [11]. Therefore, SRR holds significant potential for assisting in the visualization and assessment of lesions and vascular structures. However, its application in the pancreas is relatively new and has not yet been explored for assessing vascular invasion in PDAC.

The aim of this study was to evaluate the image quality of SRR based on T_2_WI with different slice thicknesses, as well as the feasibility and validity for preoperative assessment of vascular invasion in PDAC.

## Materials and methods

The study protocol was approved by the Institutional Review Board, and the requirement for written informed consent was waived by the Ethical Review Authority due to the retrospective nature of the study (approval number: [2021]025). All procedures were carried out in accordance with the approved guidelines.

### Patient selection

**Fig. 1:**
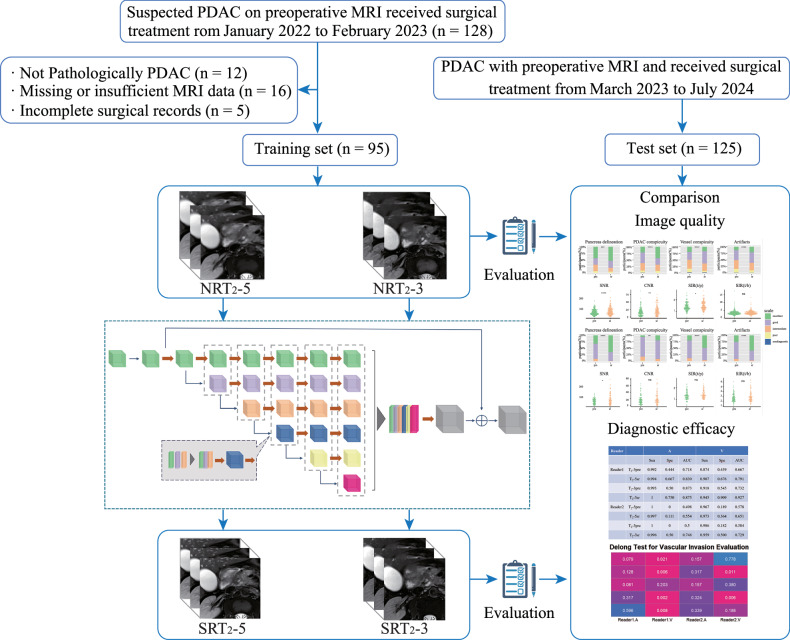
Flowchart for case enrollment and study process. PDAC, pancreatic ductal adenocarcinoma; T_2_-5, thick-slice T_2_WI; T_2_-3, thin-slice T_2_WI; NRT_2_-5, non-reconstructed thick-slice T_2_WI; NRT_2_-3, non-reconstructed thin-slice T_2_WI; SRT_2_-5, super resolution thick-slice T_2_WI; SRT_2_-3, super resolution thin-slice T_2_WI

A flowchart of the data collection and study design is shown in Fig. 1. This analysis included patients who underwent MRI within 1 month before radical surgery for suspected PDAC without neoadjuvant therapy in our hospital. The exclusion criteria were as follows: (1) missing MRI data or poor image quality; (2) missing information on tumor and vascular relationships in the surgical records; (3) not pathologically PDAC. Ultimately, 220 PDAC patients were enrolled. The training set was based on 95 cases from January 2022 to February 2023 with both thick-slice T_2_WI (T_2_-5, slice thickness ≥ 5 mm) and thin-slice T_2_WI (T_2_-3, slice thickness = 3 mm). Cases with only thick-slice T_2_WI (T_2_-5, slice thickness ≥ 5 mm, *n* = 125) from March 2023 to July 2024 were enrolled in the test set.

### Clinical and pathological information

The results of tumor-vessel contact explored by the surgeon in the surgical record were considered the gold standard in this study. Other clinical information, including patient demographics and surgical procedures, was documented. Surgical procedures were classified as pancreatoduodenectomies, partial pancreatectomies (including distal pancreatectomies, partial pancreatectomies, and tumor resections), or exploratory laparotomies.

### Imaging protocol

MRI was performed using two main 3.0-T systems (GE SIGNA Pioneer, GE Medical Systems; Magnetom Prisma, Siemens Medical Systems). Fat-suppressed T_2_WI (T_2_-fs) with different slice thicknesses are the sequences this study focuses on. The detailed MRI protocols are presented in Supplementary Table S1.

### DL‐based super-resolution technique

Non-reconstructed T_2_WI (NRT_2_) with different slice thickness (NRT_2_-3, 3 mm and NRT_2_-5, ≥ 5 mm) were based on the T_2_-fs. A previously constructed generative adversarial network (GAN)-based deep-transfer-learning network [12] was used to enhance the z-resolution, improving the image spacing to 0.1758 × 0.1758 mm. The GAN framework comprises a generator that upscales low-resolution medical volumes to high-resolution outputs, and a discriminator that distinguishes between synthetic and real images. The model was trained on millions of preprocessed low-resolution/high-resolution medical image pairs. A composite loss function combining gradient loss (edge sharpness), L1 loss (pixel-wise accuracy), and perceptual loss (feature consistency) was adopted. Details of the SRR synthesis framework are provided in the Supplementary material. The newly developed images were defined as super-resolution T_2_WI (SRT_2_) with different slice thicknesses (SRT_2_-3, 3 mm, and SRT_2_-5, ≥ 5 mm).

### Qualitative and quantitative image quality analyses

All SRT_2_ and NRT_2_ images were retrospectively reviewed by two abdominal radiologists with 3 and 9 years of experience, respectively, who were blinded to the pathological and clinical data of all patients. The SRT_2_ review was conducted two months after NRT_2_ for washout. Image evaluations and measurements were done in the 3D Slicer software (version 5.6.2, https://www.slicer.org/) [13].

Qualitative imaging quality analysis was carried out in a random order. The categories evaluated at NRT_2_ and SRT_2_ included pancreas delineation, PDAC conspicuity, vessel conspicuity, and artifacts (motion, ringing, partial volume, and susceptibility artifacts). A five-point Likert scale was used to assess each category, and the results of both raters were averaged:

1. Pancreas delineation (0 = nondiagnostic, 1 = poor, 2 = fair delineation but with margin blurring, 3 = good with sharp margins, and 4 = excellent sharpness and clear visualization of the pancreatic duct);
2. PDAC conspicuity (0 = nondiagnostic, 1 = poor or merely recognizable, 2 = intermediate, 3 = good, and 4 = excellent);
3. vessel conspicuity (0 = nondiagnostic, 1 = poor or merely recognizable, 2 = intermediate, 3 = good, and 4 = excellent);
4. ghosting, motion or susceptibility artifacts (0 = severe, 1 = poor, 2 = moderate, 3 = mild, and 4 = absent)

The signal intensity (SI) of the PDAC lesion (SI_tumor_), pancreatic parenchyma (SI_pan_), and spine erector muscle (SI_background_), as well as the standard deviation (SD) of the spine erector muscle (SD_background_) were assessed. This involved manually outlining regions of interest (ROIs) on axial images, encompassing as much of the tumor as feasible. Careful attention was paid to avoid areas featuring cystic degeneration, necrosis, adjacent vessels, or dilated ducts during ROI selection. SI_tumor_ and SI_background_ were measured at the same slice of the PDAC lesion and ipsilateral spine erector muscle (Fig. S1A). The inclusion of adipose tissue was avoided. The attenuation values for pancreatic parenchyma were measured three times at different slices of the head, neck, body, or tail, carefully avoiding tumor involvement (Fig. S1B–D). SI_pan_ is the average of pancreatic parenchyma measurements, subsequently utilized for further analysis.

The tumor signal-to-noise ratio (SNR), contrast-to-noise ratio (CNR), and signal-intensity ratio (SIR) [14] were formulated and computed according to the following equation:

$${{{\rm{SNR}}}}={{{{\rm{SI}}}}}_{{{{\rm{tumor}}}}}/{{{{\rm{SD}}}}}_{{{{\rm{background}}}}}$$

$${{{\rm{CNR}}}}=|{{{{\rm{SI}}}}}_{{{{\rm{tumor}}}}}-{{{{\rm{SI}}}}}_{{{{\rm{pan}}}}}|/{{{{\rm{SD}}}}}_{{{{\rm{background}}}}}$$

$${{{{\rm{SIR}}}}}_{{{{\rm{t}}}}/{{{\rm{p}}}}}={{{{\rm{SI}}}}}_{{{{\rm{tumor}}}}}/{{{{\rm{SI}}}}}_{{{{\rm{pan}}}}}$$

$${{{{\rm{SIR}}}}}_{{{{\rm{t}}}}/{{{\rm{b}}}}}={{{{\rm{SI}}}}}_{{{{\rm{tumor}}}}}/{{{{\rm{SI}}}}}_{{{{\rm{background}}}}}$$

### Vessel invasion analyses

The relationships between the tumor and main arteries (including the celiac trunk artery, hepatic artery, and superior mesenteric artery), and the portal vein-superior mesenteric vein were recorded. The analysis included circumferential tumor contact (less than or equal to 180° vs more than 180°), vessel narrowing (change in the vessel caliber), and contour irregularity (interruption or obstruction of the vessel wall). The invasion of each vessel was determined using the following criteria: (1) circumferential tumor contact > 180°, (2) vessel narrowing, and (3) contour irregularity. All cases were assessed by two radiologists (blind to the case name or the vascular invasion result) on the above three points. After the evaluation, the senior radiologist read the results according to the criteria and performed statistical analysis. The efficacy of vascular assessment was calculated and compared for each group.

### Test set evaluation

SRR was applied to cases in the test set. To evaluate the improvement in diagnostic accuracy for vascular invasion, the diagnostic efficacy of vascular invasion assessments was calculated and compared before and after SRR.

### Statistical analysis

SPSS (version 25.0; IBM Corp.) and R (version 4.2.2) were used for the analysis. Continuous variables were evaluated using the Kolmogorov–Smirnov normality test. Means and SDs were used to describe the normal continuous variables. Median and interquartile ranges are used to describe non-normal variables. The *t*-test was performed for normally distributed data, and the Kruskal–Wallis test was performed for non-normally distributed data. Categorical variables were analyzed using the χ2 or Fisher’s exact test. Friedman's non-parametric test was used to analyze non-normal variables. Multiple comparisons between groups were then performed using the Quade test. To analyze inter-observer agreement, the second-order agreement coefficient (AC2) was used for categorical variables and interclass correlation coefficients (ICCs) for continuous variables. The interobserver agreement was graded as follows: 0–0.20, slight; 0.21–0.40, fair; 0.41–0.60, moderate; 0.61–0.80, substantial; and 0.81–1.00, almost perfect. Receiver operating characteristic (ROC) curves were used to evaluate the predictive efficacy of each group and the criteria of sensitivity (Sen), specificity (Spe), and area under the ROC curve (AUC). The Delong test and Net reclassification improvement index (NRI) test were used to compare the accuracy of the different evaluation methods. Decision curve analysis (DCA) was applied to quantify the net benefits of the models. All differences were considered statistically significant at *p* < 0.05.

## Result

### Clinical characteristics of participants

A total of 95 patients with a mean age of 59.47 years (SD, 11.02) were included in the training set and 125 patients with a median age of 64 years (range, 54–70) were included in the test set (Fig. 1). Complete clinical information of both groups is given in Table 1.

**Table 1:** Clinical characteristics of enrolled participants

Variable	Training set	Test set
No. of participants	95	125
Age (y)	59.47 (±11.02)	64 (54,70)
Sex (male)	63 (66.3)	75 (60.0)
CEA	3.43 (2.15, 6.12)	2.90 (2.03, 4.53)
CA125	17.80 (11.40, 29.90)	17.40 (12.40, 31.30)
CA199	169.68 (41.48, 1307.14)	138.37 (22.36, 987.54)
Location (head/neck)	63 (66.3)	87 (69.6)
Surgical Procedure		
Pancreaticoduodenectomy	71 (74.7)	90 (72.0)
Distal pancreatectomy	17 (17.9)	32 (25.6)
Total pancreatectomy	2 (2.1)	0 (0.0)
Exploration and other palliative procedures	5 (5.3)	3 (2.4)
Vascular invasion		
Vein	19 (20.0)	12 (9.6)
Arteries	3 (3.2)	4 (3.2)
Arteries and veins	5 (5.3)	3 (2.4)

*CEA* carcinoembryonic antigen, *CA125* carbohydrate antigen 125, *CA199* carbohydrate antigen 199

### Image quality evaluation

#### Comparison of qualitative image evaluations

For the qualitative assessment, before and after reconstruction were performed within the T_2_-5 and T_2_-3 groups. Compared with NRT_2_-5, SRT_2_-5 more frequently achieved an averaged reader score 4 or higher for pancreas delineation (51% [122 of 220 participants] vs 69% [152 of 220]; *p* < 0.001), lesion conspicuity (50% [110 of 220] vs 56% [123 of 220]; *p* < 0.001), vessel conspicuity (35% [77 of 220] vs 51% [113 of 220]; *p* < 0.001), and artifacts (38% [83 of 220] vs 49% [108 of 220]; *p* < 0.001).

Compared with NRT_2_-3, SRT_2_-3 more frequently achieved an averaged reader score 4 or higher for pancreas delineation (76% [72 of 95 participants] vs 98% [93 of 95]; *p* < 0.01), lesion conspicuity (77% [73 of 95] vs88% [84 of 95]; *p* < 0.001), vessel conspicuity (94% [89 of 95] vs 99% [94 of 95] ; *p* < 0.001), and artifacts (96% [91 of 95] vs 87% [83 of 95] ; *p* < 0.001).

Quade’s test focused on differences between T_2_-3 and T_2_-5. Regardless of before and after reconstruction, T_2_-3 showed better pancreatic and vessel delineation, and fewer artifacts than T_2_-5 (*p* < 0.05). The difference in showing tumors before reconstruction was a strong tendency towards statistical significance (*p* = 0.052), while the difference after reconstruction was not significant. There was no significant difference between SRT_2_-5 and NRT_2_-3 in terms of pancreas delineation and tumor conspicuity. The detailed Quade test results are provided in Table 2.

**Table 2:** Quade test for multiple comparisons between groups

Comparison	Pancreas delineation	PDAC conspicuity	Vessel conspicuity	Artifacts	SNR	CNR	SIR(t/p)	SIR(t/b)
NRT_2_-5 vs SRT_2_-5	0.00881	0.002	0.006	< 0.001	0.03	0.511	< 0.001	0.564
NRT_2_-3 vs SRT_2_-3	0.00015	0.042	0.003	< 0.001	0.61	0.634	< 0.001	0.003
NRT_2_-5 vs NRT_2_-3	0.00296	0.052	< 0.001	< 0.001	0.77	0.634	1	< 0.001
SRT_2_-5 vs SRT_2_-3	< 0.001	0.331	< 0.001	< 0.001	0.77	0.634	1	0.167
SRT_2_-5 vs NRT_2_-3	0.64692	0.331	0.003	< 0.001	0.17	0.049	< 0.001	< 0.001
NRT_2_-5 vs SRT_2_-3	< 0.001	< 0.001	< 0.001	< 0.001	0.21	0.821	< 0.001	0.564

*NRT*_*2*_*-5* non-reconstructed thick-slice T_2_WI, *NRT*_*2*_*-3* non-reconstructed thin-slice T_2_WI, *SRT*_*2*_*-5* super resolution thick-slice T_2_WI, *SRT*_*2*_*-3* super resolution thin-slice T_2_WI, *SNR* signal-to-noise ratio, *CNR* contrast-to-noise ratio, *SIR(t/p)* signal-intensity ratio between the tumor and pancreas, *SIR(t/b)* signal-intensity ratio between the tumor and background

**Fig. 2:**
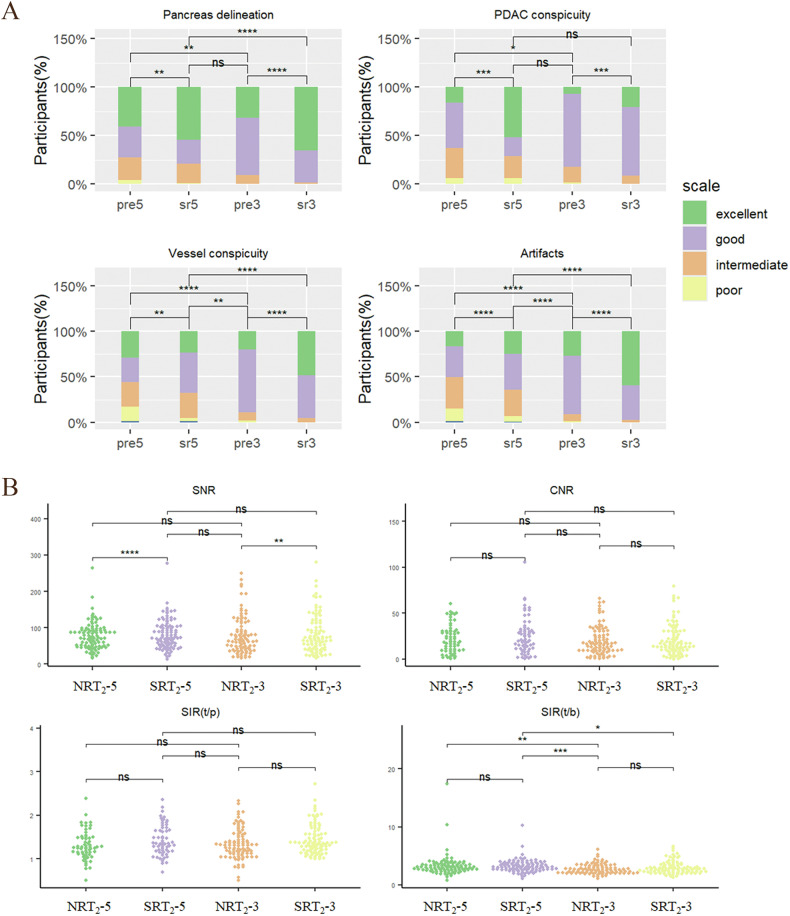
Stacked bar charts and dot plots for the qualitative and quantitative image evaluation stratified by slice thickness (T_2_-3, 3 mm and T_2_-5, ≥ 5 mm) and sequence type (non-reconstructed T_2_WI, NRT_2_; super-resolution T_2_WI, SRT_2_). **A** Stacked bar charts show qualitative 5-point Likert scale scores averaged between the two readers. Qualitative ratings of pancreas delineation, PDAC conspicuity, vessel conspicuity, and artifacts were improved after super-resolution reconstruction (SRR). **B** Dot plots show comparisons of quantitative evaluation metrics, including tumor SNR, CNR, tumor-to-pancreas SIR_t/p_, and tumor-to-background SIR_t/b_. The SRT_2_ showed higher SNR in comparison with NRT_2_ in T_2_-5 and T_2_-3 groups. The SIR_t/b_ of T_2_-5 was higher than T_2_-3. Ns, *p* > 0.05; ^*^p < 0.05; ^**^p < 0.01; ^***^p < 0.001; ^****^p < 0.0001

**Fig. 3:**
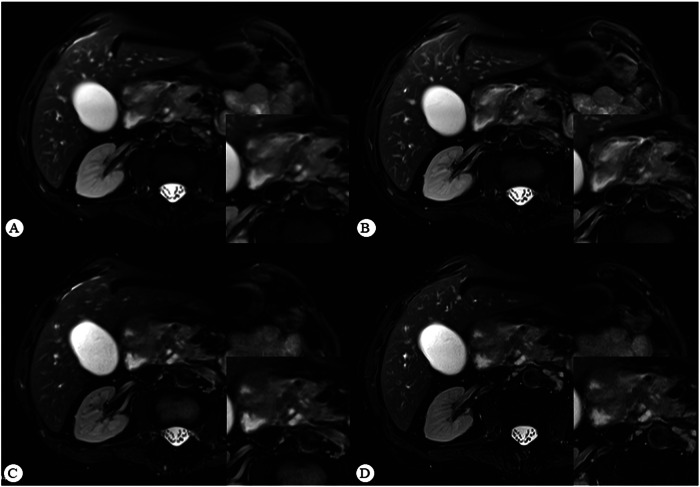
Comparison of image quality before and after SRR. SRT_2_-5 (**B**) and SRT_2_-3 (**D**) show clearer details of organ edges and internals than NRT_2_-5 (**A**) and NRT_2_-3 (**C**)

**Fig. 4:**
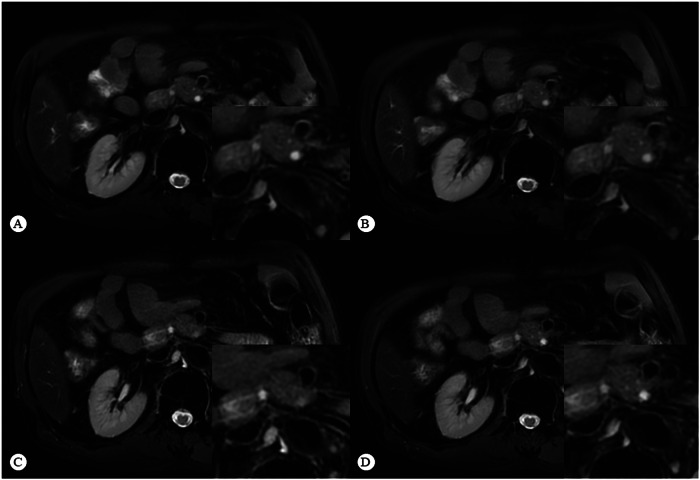
Comparison of vascular evaluation before and after SRR. SRT_2_-5 (**B**) more clearly shows the superior mesenteric vein wall invasion than NRT_2_-5 (**A**). NRT_2_-3 (**C**) shows a suspicious cusp sign in the superior mesenteric vein. SRT_2_-3 (**D**) shows the vessel wall invasion more clearly, which increases the diagnostic confidence

Intraclass correlation coefficients ranged from substantial to almost perfect agreement across all qualitative categories assessed (range, 0.78–0.84 [95% CI: 0.72, 0.87]) (Table 3). The distribution of scores for each sequence type is shown in Fig. 2. Representative participant images are shown in Figs. 3 and 4.

**Table 3:** Qualitative and quantitative image evaluation ratings based on the average score assigned by two readers

Category	NRT_2_-5	SRT_2_-5	NRT_2_-3	SRT_2_-3	Friedman	Difference^#^	AC2/ICC^*^
Pancreas delineation	4 (3, 5)	4 (3, 5)	4 (4, 5)	5 (4, 5)	< 0.001	0.386	0.84 (0.81, 0.87)
PDAC conspicuity	4 (3, 4)	4 (3, 5)	4 (4, 4)	4 (4, 4)	< 0.001	0.6411	0.84 (0.81, 0.87)
Vessel conspicuity	3 (2, 4)	4 (3, 4)	4 (4, 4)	4 (4, 5)	< 0.001	0.5951	0.78 (0.72, 0.83)
Artifacts	3 (2.5, 4)	4 (3, 4)	4 (4, 4)	5 (4, 5)	< 0.001	0.581	0.79 (0.73, 0.85)
SNR	72.46 (50.23, 94.86)	74.18 (51.77, 104.76)	64.15 (40.67, 100.96)	69.49 (42.41, 112.73)	0.01	0.1243	0.76 (0.49, 0.89)
CNR	19.04 (9.63, 30.44)	20.64 (11.03, 31.81)	16.44 (9.31, 29.25)	16.20 (9.81, 30.36)	0.065	0.8523	0.87 (0.70, 0.94)
SIR(t/p)	1.30 (0.33)	1.42 (0.35)	1.31(1.12, 1.54)	1.35 (1.19, 1.62)	< 0.001	0.3103	0.82 (0.76, 0.86)
SIR(t/b)	3.00 (2.55, 3.55)	3.03 (2.59, 3.79)	2.58 (2.09, 3.26)	2.73 (2.20, 3.45)	< 0.001	0.0296	0.87 (0.83, 0.90)

*NRT*_*2*_*-5* non-reconstructed thick-slice T_2_WI, *NRT*_*2*_*-3* non-reconstructed thin-slice T_2_WI, *SRT*_*2*_*-5* super resolution thick-slice T_2_WI, *SRT*_*2*_*-3* super resolution thin-slice T_2_WI, *SNR* signal-to-noise ratio, *CNR* contrast-to-noise ratio, *SIR(t/p)* signal-intensity ratio between the tumor and pancreas, *SIR(t/b)* signal-intensity ratio between the tumor and background

^#^ *p*-value for the comparison of variations in T_2_-3 and T_2_-5 before and after SR reconstruction

* The AC2 was used for categorical variables, and ICCs for continuous variables

### Comparison of quantitative image evaluations

All indicators except CNR were significantly different between all groups. SRR significantly improved the SNR in both groups (T_2_-5, 72.46 vs 74.18; T_2_-3, 64.15 vs 69.49; *p* < 0.05). CNR, SIR_t/p_, and SIR_t/b_ were not significantly affected. SRR significantly improved SIR_t/b_ for T_2_-3 more than for T_2_-5. Further cross-group analyses showed that SIR_t/b_ was significantly higher in T_2_-5 than in T_2_-3 before reconstruction (*p* < 0.001), while no significant difference was found in all quantitative evaluations after reconstruction. SRT_2_-5 showed higher CNR, SIR_t/p_, and SIR_t/b_ than NRT_2_-3. The results of the quantitative assessment are shown in Fig. 2 and Tables 2 and 3.

The ICC for intrareader reproducibility of quantitative measurements was substantial for apparent SNR (0.76 [95% CI: 0.49, 0.89]) and almost perfect for apparent CNR, SIR_t/p_, and SIR_t/b_ (0.82–0.87).

### Vascular invasion evaluations

**Fig. 5:**
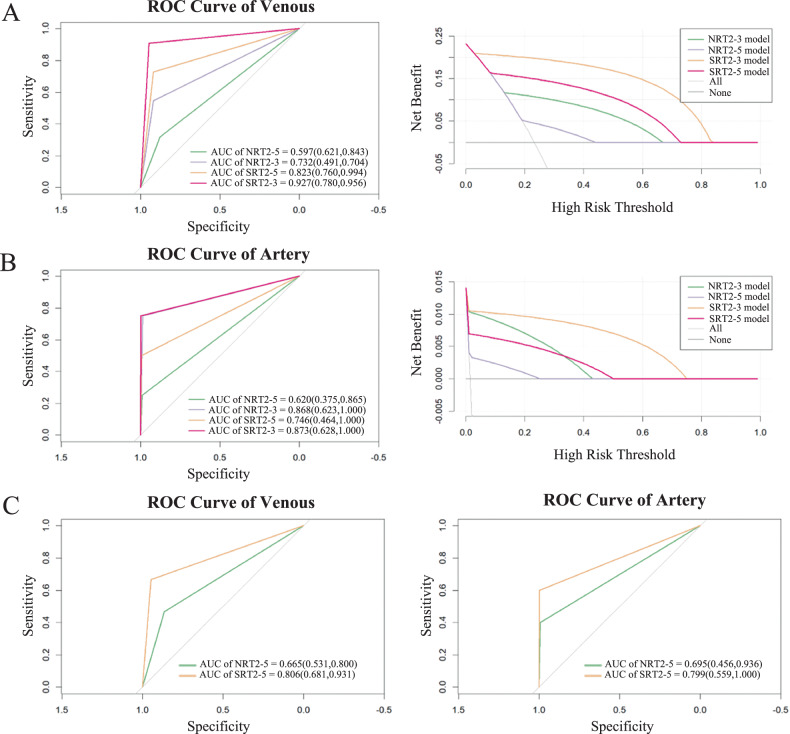
Efficacy of vascular invasion assessment in training and test sets. **A** ROC curve and DCA for venous assessment. **B** ROC curves and DCA for arterial assessment. **C** ROC curves for the test set

By both radiologists, SRT_2_-3 showed the highest overall AUC value and Spe for venous invasion assessments (Fig. 5). The senior reader showed a higher AUC value than the junior reader in assessing arterial and venous invasion. Before reconstruction, NRT_2_-3 performs better than NRT_2_-5 in diagnosing venous invasion (AUC, 0.732 vs 0.597, *p* = 0.0187). Reconstruction significantly improved the diagnosis of venous invasion in two readers (*p* < 0.05). The difference in diagnostic efficacy of SRT_2_-5 and SRT_2_-3 in diagnosing venous invasion was not significant. There were no significant differences between SRT_2_-5 and NRT_2_-3 in the assessment. The diagnostic improvement of SRT_2_-5 over NRT_2_-5 is higher than that of SRT_2_-3 over NRT_2_-3. The disparity between SRT_2_-3 and SRT_2_-5 is smaller than that of NRT_2_-3 and NRT_2_-5. SRR narrowed the gap between T_2_-5 and T_2_-3. Neither slice thickness nor reconstruction had a significant effect on the diagnosis of arterial invasion. The results of vascular evaluation and comparison are shown in Supplementary Table S2 and Table 4. Subgroup analysis showed no significant difference between the two scanners in assessing vessel invasion (Supplementary Table S3).

**Table 4:** Comparison of vascular invasion evaluation

	NRI test				Delong test	
	Arteries		Venous		Arteries	Venous
Training set	NRI (95% CI)	*p*-value	NRI (95% CI)	*p*-value	*p*-value	*p*-value
SRT_2_-5 vs NRT_2_-5	0.2536 (−0.171, 0.6781)	0.24173	0.4502 (0.233, 0.6674)	< 0.001	0.3106	< 0.001
SRT_2_-3 vs NRT_2_-3	0.0107 (−0.0013, 0.0227)	0.08162	0.3001 (0.0691, 0.5311)	0.01087	0.08216	0.01276
NRT_2_-5 vs NRT_2_-3	−0.4964 (−0.9866, −0.0063)	0.04713	−0.2684 (−0.4921, −0.0446)	0.01872	0.08555	0.02147
SRT_2_-5 vs SRT_2_-3	−0.2536 (−1.0662, 0.559)	0.54081	−0.1183 (−0.3702, 0.1336)	0.35731	0.5964	0.3683
NRT_2_-3 vs SRT_2_-5	−0.2429 (−1.0555, 0.5697)	0.558	0.1818 (−0.0673, 0.4309)	0.15254	0.6119	0.1617
Test set						
SRT_2_-5 vs NRT_2_-5	0.2054 (−0.1454, 0.5562)	0.25108	0.281 (0.0715, 0.4921)	0.00863	0.3046	0.011

*NRT*_*2*_*-5* non-reconstructed thick-slice T_2_WI, *NRT*_*2*_*-3* non-reconstructed thin-slice T_2_WI, *SRT*_*2*_*-5* super resolution thick-slice T_2_WI, *SRT*_*2*_*-3* super resolution thin-slice T_2_WI, *NRI* net reclassification improvement index, *CI* confidence interval

## Discussion

For patients with PDAC, complete surgical resection is crucial for long-term survival, with vascular invasion being an important influencing factor. Therefore, accurate preoperative evaluation is essential for developing an effective therapeutic plan. This study used a DL-based SRR technique for both thin-slice and thick-slice T_2_WI, confirming that both thin-slice T_2_WI and SRT_2_ offer superior image quality and diagnostic efficacy compared to thick-slice T_2_WI and NRT_2_. This indicates that both thin-slice T2 and SRR have the potential to help PDAC achieve precise preoperative assessment and make better treatment decisions for patients.

The results of this study indicated that T_2_-3 yields better image quality with fewer artifacts than T_2_-5. MRI slice thickness plays a crucial role in visualizing tissue structures. Thin-slice T_2_WI enhances the clarity of vessel wall structure and integrity, supporting a more precise assessment of vascular invasion in PDAC. Matsumoto et al [7] showed that thin-slice T_2_WI for the rectum provided better image quality than thick-slice imaging. Similarly, in pancreatic imaging, 3-mm T_2_WI produced higher image quality compared to 6-mm T_2_WI [15]. However, Kim et al [16] reported that in prostate imaging, the image quality and diagnostic accuracy of 2-mm T_2_WI were actually inferior to those of 3-mm T_2_WI. These results imply that slice thickness and image quality do not have a simple inverse correlation, underscoring the need for a comprehensive evaluation to determine the optimal slice thickness.

The results of this study demonstrated that SRR significantly improves pancreas delineation, lesion conspicuity, vessel conspicuity, and reduces artifacts. DL techniques are increasingly used in medical imaging, with DL-based SRR proving effective for enhancing diagnostic accuracy by improving image quality and providing more diagnostic information [6, 7, 12, 17]. In abdominal imaging, SRR can effectively enhance image quality, lesion conspicuity, and diagnostic confidence for both pre- and post-contrast T_1_-weighted imaging [18]. SRR also improved the SNR and CNR of images in previous studies [6, 7]. But in this study, SRR remarkably improved only SNR for T_2_-5 and T_2_-3 (Fig. 3). After controlling for multiple comparisons, the SIR_t/p_ of T_2_-5 and T_2_-3 also showed significant improvement. Additionally, SRT_2_-5 exhibited higher CNR, SIR_t/p_ and SIR_t/b_ than NRT_2_-3 (Table 2). Although SRR did not significantly outperform T_2_-3 in terms of image quality improvement for T_2_-5, the image quality of SRT_2_-5 was comparable or even better than that of NRT_2_-3. This improved tumor-to-pancreas contrast in SRT_2_-5 images likely contributed to the enhanced visualization of the tumor relative to adjacent structures and higher subjective image quality scores.

This study focused on T_2_WI and found that T_2_-3 was more effective in diagnosing vascular invasion than T_2_-5. Thin-slice high-resolution MRI enhances lesion detection and diagnosis across a range of diseases [14, 19, 20]. High-resolution T_2_WI (with slice thickness ≤ 3 mm) is important for the staging of rectal cancer and allows assessment of mesorectal fascia involvement [21]. Our previous study also demonstrated that high-resolution MRI is particularly useful for evaluating vascular invasion in PDAC [20]. After further applying SRR, SRT_2_ exhibited higher Sen and Spe in detecting vascular invasion compared to NRT_2_, especially for venous invasion. The combination of SRR and thin-slice MRI yielded better diagnostic outcomes for assessing vascular invasion. Both SRT_2_ and NRT_2_ showed excellent specificity and inadequate sensitivity in evaluating vessel invasion. The main improvement in SRR is the diagnostic sensitivity, which means better detection of patients with vascular invasion. This improvement prevents patients from undergoing unnecessary surgeries and allows for better planning of treatment programs. There was no significant difference in the diagnostic efficacy between SRT_2_-5 and NRT_2_-3. Therefore, SRR of thick-slice T_2_WI also presents an effective option for improving diagnostic accuracy, particularly under constrained conditions. This method is scanner-independent, non-disruptive to clinical workflow, and offers the potential advantage of image quality enhancement without prolonging acquisition time.

When comparing the assessments of the two readers, the impact of slice thickness was not significant for the junior reader, but SRR led to significant improvements (Supplementary Table S2). While SRR enhanced image quality, Sen, and Spe for evaluating venous invasion, it did not similarly improve the accuracy of arterial invasion diagnosis (Table 4). For the senior reader, the effect of slice thickness on diagnosing arterial invasion approached significance, but SRR showed no significant impact. A similar trend was observed in the junior readers’ assessment. The reader assessments demonstrated high Spe but comparatively lower Sen in evaluating vascular invasion, particularly among arterial assessment and the junior reader. This may be attributed to several factors: First, potential selection bias due to the low true-positive rate (especially arterial invasion) in the included cases. Second, when encountering equivocal imaging findings, the junior reader may choose to be more conservative. Third, a less accurate interpretation of diagnostic features by the junior reader is also a possible reason. Future studies with larger sample sizes are needed to better evaluate the accuracy of arterial invasion assessments.

Several limitations associated with the present study warrant mention. First, the sample size and true-positive rate in this single-center study were limited, which may have resulted in selection bias. Subgroup analysis of scanner-related variability showed no significant difference between the two groups in vessel evaluation results (Supplementary Table S3). Further multicenter, prospective studies with a large number of cases are needed to determine the usefulness of improving clinical determinations and the impact of scanners. Second, ROIs were manually positioned for the quantitative measurement, while volumetric measurement of the entire lesion would be preferable for a more accurate analysis in a future study. Third, only patients at their first medical visit were considered in this study. Future investigations should consider the resectability assessment of PDAC after neoadjuvant chemotherapy to provide a more comprehensive evaluation.

## Conclusion

Thin-slice T_2_WI and SRR can effectively improve image quality and diagnostic efficacy for assessing venous invasion in PDAC, enabling more precise preoperative vascular evaluations. Additionally, applying SRR to thick-slice T_2_WI offers a time-efficient alternative, delivering diagnostic results comparable to those of thin-slice T_2_WI while reducing scanning duration.

## Supplementary information

ELECTRONIC SUPPLEMENTARY MATERIAL

## Data Availability

The datasets generated and analyzed during the current study are not publicly available due to participant privacy and ethical restrictions, but are available from the corresponding author on reasonable request.

## Abbreviations

AC2: Second-order agreement coefficient
AUC: Area under the ROC curve
CNR: Contrast-to-noise ratio
DCA: Decision curve analysis
DL: Deep learning
GAN: Generative adversarial network
ICC: Interclass correlation coefficients
NRI: Net reclassification improvement index
NRT2: Non-reconstructed T2WI
NRT2-3: Non-reconstructed T2WI with slice thickness = 3 mm
NRT2-5: Non-reconstructed T2WI with slice thickness ≥ 5 mm
PDAC: Pancreatic ductal adenocarcinoma
ROC: Receiver operating characteristic
ROI: Region of interest
SD: Standard deviation
Sen: Sensitivity
SI: Signal intensity
SIR: Signal-intensity ratio
SNR: Signal-to-noise ratio
Spe: Specificity
SRR: Super-resolution reconstruction
SRT2: Super-resolution T2WI
SRT2-3: Super-resolution T2WI with slice thickness = 3 mm
SRT2-5: Super-resolution T2WI with slice thickness ≥5 mm
T2-3: T2-weighted imaging with slice thickness = 3 mm
T2-5: T2-weighted imaging with slice thickness ≥5 mm
T2WI: T2-weighted imaging
