# Supplementary material for: Thin-slice T2-weighted images and deep-learning-based super-resolution reconstruction: improved preoperative assessment of vascular invasion for pancreatic ductal adenocarcinoma
Source: Insights Imaging. 2025 Jun 30;16:144. doi: 10.1186/s13244-025-02022-5 (PMC12209124; doi:10.1186/s13244-025-02022-5)
Supplement: Supplementary file 1 — ELECTRONIC SUPPLEMENTARY MATERIAL [file 13244_2025_2022_MOESM1_ESM.pdf]

Thin-slice T2-Weighted Images and Deep-Learning-Based Super-Resolution Reconstruction: Improved Preoperative Assessment of Vascular Invasion for Pancreatic Ductal Adenocarcinoma

ELECTRONIC SUPPLEMENTARY MATERIAL

Generative Adversarial Network (GAN)-based Deep-transfer-learning Network

The 3D super-resolution reconstruction technique provided in our method utilizes a GAN as its basic architecture. GANs are a type of deep learning model that consists of two networks: a generator network and a discriminator network. The generator network generates high-resolution images from low-resolution images, while the discriminator network distinguishes between real and generated images. The two networks are trained in an adversarial manner, where the generator network tries to generate images that can fool the discriminator network, and the discriminator network tries to distinguish between real and generated images. This adversarial training process helps the generator network to learn the mapping between low-resolution and high-resolution images.

The dataset used to train the 3D super-resolution reconstruction technique consists of millions of medical images. The images are preprocessed to remove noise and artifacts and to normalize the intensity values. The images are then divided into low-resolution and high-resolution pairs, where the low-resolution images are obtained by downsampling the high-resolution images. The pairs are used to train the GAN model.

The loss function used in the GAN model consists of three components: gradient loss, L1 loss, and perceptual loss. The gradient loss encourages the generated images to have similar gradient values as the high-resolution images.

The L1 loss measures the pixel-wise difference between the generated and high-resolution images. The perceptual loss measures the difference between the feature representations of the generated and high-resolution images obtained from a pre-trained deep learning model. The combination of these loss functions helps to ensure that the generated images are visually similar to the high-resolution images.

The 3D super-resolution reconstruction technique we provided has shown promising results in improving the spatial resolution of medical images. For example, it can increase the spatial resolution by 4 times while maintaining the original image size. This means that a pixel volume of 1x1x1mm can be transformed into 1x1x0.25mm. The technique has been evaluated on various medical imaging modalities such as CT, MRI, and ultrasound, and has shown significant improvement in image quality and spatial resolution. The technique has also been compared with other state-of-the-art super-resolution reconstruction techniques and has shown superior performance.

Supplementary Table S1. MRI protocols

T_2-fs	TR (ms)	TE (ms)	FOV (mm)	Matrix (mm)	Slice thickness (mm)	FA (°)
Scanner 1	4765	77	380	384×288	5	103
	4437	78	350	384×288	3	103
Scanner 2	10588	68	340	320×320	5	111
	7500	62	320	320×320	3	111

T_2-fs, Fat suppressed T_2WI; TR, repetition time; TE, echo time; FOV, field of view; FA, flip angle.

Supplementary Table S2. Evaluation of Vascular Invasion by Readers

Reader		Arteries			Venous		
		Sen	Spe	AUC	Sen	Spe	AUC
Reader1	NRT_2-5	0.250	0.989	0.620	0.318	0.877	0.597
	SRT_2-5	0.500	0.993	0.746	0.727	0.918	0.823
	NRT_2-3	0.750	0.986	0.868	0.545	0.918	0.732
	SRT_2-3	0.750	0.996	0.873	0.818	0.945	0.882
Reader2	NRT_2-5	0	1	0.498	0.189	0.967	0.578
	SRT_2-5	0.111	0.997	0.554	0.364	0.973	0.651
	NRT_2-3	0	1	0.50	0.182	0.986	0.584
	SRT_2-3	0.50	0.996	0.748	0.500	0.959	0.729

Reader1, senior reader; Reader2, junior reader; NRT_2-5, non-reconstructed thick-slice T_2WI; NRT_2-3, non-reconstructed thin-slice T_2WI; SRT_2-5, super resolution thick-slice T_2WI; SRT_2-3, super resolution thin-slice T_2WI; Sen, sensitivity; Spe, specificity; AUC, area under the curve.

Supplementary Table S3. Subgroup analysis based on different Scanner

		Scanner 1	Scanner 2	*P*
NRT_2-5	Pancreas delineation	4.5(3.75, 5)	4(3, 4)	0.017
	PDAC conspicuity	3.5(3, 4)	4(3, 4)	0.456
	Vessel conspicuity	3(2, 4)	3(2, 4)	0.550
	Artifacts	3.5(3, 4)	3(2, 4)	0.016
	SNR	45.92(38.24, 55.38)	87.26(67.05, 101.92)	<0.0001
	CNR	19.28(10.48, 25.05)	19.04(9.63, 32.43)	0.421
	SIR(t/p)	1.27(1.23, 1.50)	1.25(1.09, 1.47)	0.234
	SIR(t/b)	3.48(2.84, 4.00)	2.85(2.36, 3.32)	0.0004
	AUC of Venous Evaluation	0.563	0.629	0.490
	AUC of Arteries Evaluation	0.494	0.662	0.464
SRT_2-5	Pancreas delineation	5(5, 5)	4(3, 5)	0.0001
	PDAC conspicuity	5(5, 5)	3(3, 4)	<0.0001
	Vessel conspicuity	4(3, 4)	4(3, 4)	0.907

	Artifacts	4(3, 4.25)	3(3, 4)	0.119
	SNR	45.09(39.91, 61.13)	91.78(68.42, 120.68)	<0.0001
	CNR	18.77(10.46, 28.08)	23.04(11.57, 33.03)	0.336
	SIR(t/p)	1.47(1.30, 1.77)	1.30(1.11, 1.64)	0.105
	SIR(t/b)	3.56(3.13, 4.16)	2.92(2.50, 3.42)	0.0006
	AUC of Venous Evaluation	0.875	0.801	0.482
	AUC of Arteries Evaluation	0.494	0.831	1.000
NRT_2-3	Pancreas delineation	4(3.75, 4)	4(4, 5)	0.0003
	PDAC conspicuity	3.5(3, 4)	4(4, 4)	<0.0001
	Vessel conspicuity	4(3, 4)	4(4, 4)	0.0001
	Artifacts	4(4, 4)	4(4, 5)	0.0009
	SNR	34.19(23.83, 41.03)	80.59(61.76, 124.76)	<0.0001
	CNR	9.85(7.46, 17.33)	19.09(10.36, 33.72)	0.0023
	SIR(t/p)	1.46(1.30, 1.81)	1.23(1.06, 1.39)	0.0013
	SIR(t/b)	3.38(2.86,	2.30(1.99,	<0.0001

		4.02)	2.73)	
	AUC of Venous Evaluation	0.850	0.667	0.106
	AUC of Arteries Evaluation	0.994	0.827	0.678
SRT_2-3	Pancreas delineation	5(4, 5)	5(4, 5)	0.522
	PDAC conspicuity	4(4, 4)	4(4, 4)	0.530
	Vessel conspicuity	4.5(4, 5)	4(4, 5)	0.177
	Artifacts	5(4, 5)	4(4, 5)	0.196
	SNR	33.16(24.83, 41.54)	87.43(64.73, 132.96)	<0.0001
	CNR	13.24(7.03, 15.95)	19.29(11.05, 34.63)	0.0028
	SIR(t/p)	1.56(1.36, 1.87)	1.31(1.18, 1.47)	0.0024
	SIR(t/b)	3.88(3.20, 4.87)	2.46(2.13, 2.89)	<0.0001
	AUC of Venous Evaluation	0.875	0.891	0.871
	AUC of Arteries Evaluation	0.994	0.833	1.000

NRT_2-5, non-reconstructed thick-slice T_2WI; NRT_2-3, non-reconstructed

thin-slice T_2WI; SRT_2-5, super resolution thick-slice T_2WI; SRT_2-3, super resolution thin-slice T_2WI; SNR, signal-to-noise ratio; CNR, contrast-to-noise ratio; SIR(t/p), signal-intensity ratio between the tumor and pancreas; SIR(t/b), signal-intensity ratio between the tumor and background; AUC, area under the curve.

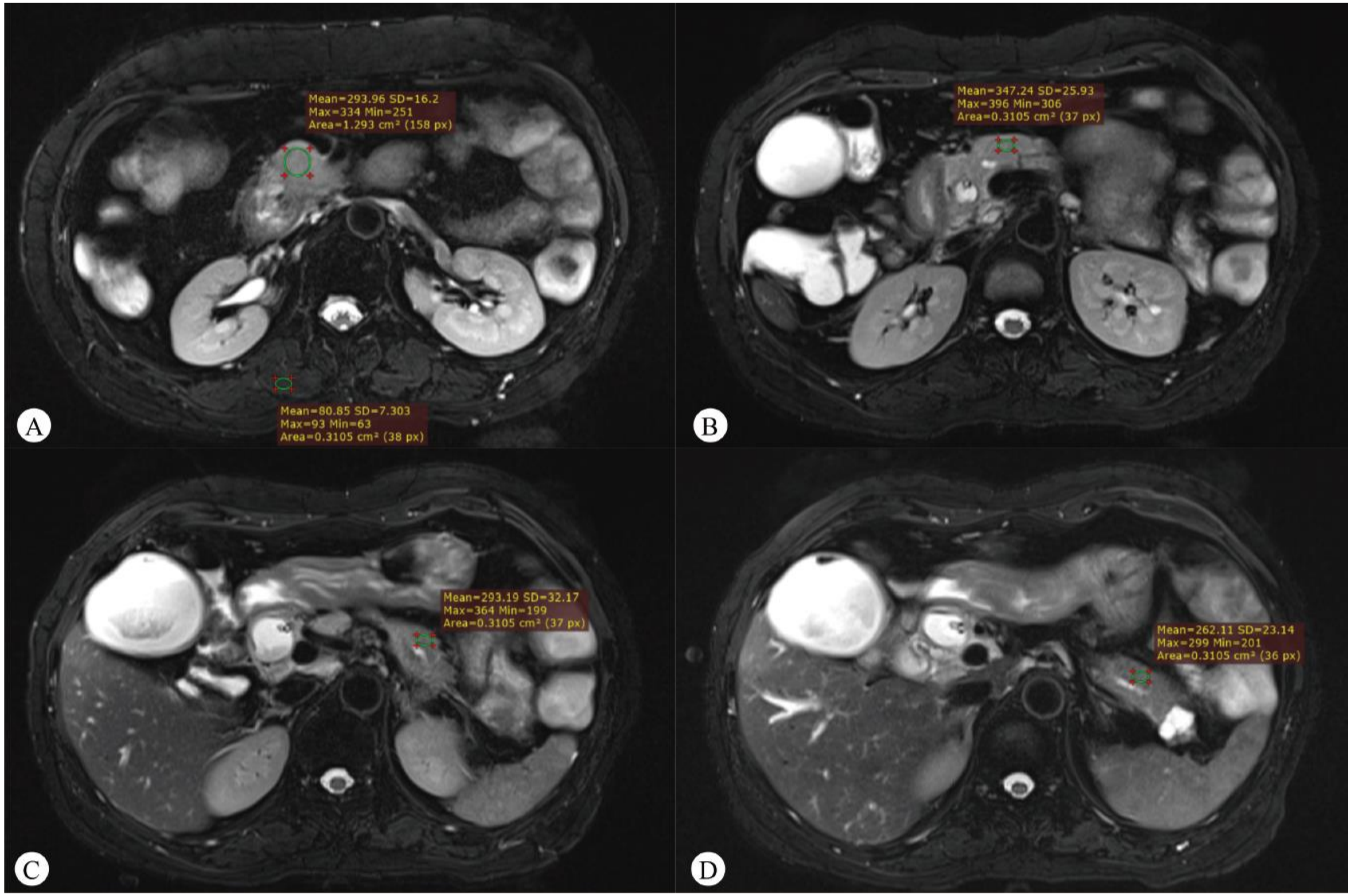

Figure S1 A. SI_{tumor} and $SI_{background}$ were measured at the same slice of the PDAC lesion and ipsilateral spine erector muscle. **Figure S1 B-D** Attenuation values for pancreatic parenchyma were measured three times at different slices of head, neck, body or tail, carefully avoiding tumor involvement.
